# Femoral Neck Anteversion and Neck Shaft Angles: Determination and their Clinical Implications in Fetuses of Different Gestational Ages

**DOI:** 10.5704/MOJ.1507.009

**Published:** 2015-07

**Authors:** AD Souza, VH Ankolekar, S Padmashali, A Das, ASD Souza, M Hosapatna

**Affiliations:** Department of Anatomy, Kasturba Medical College, Manipal, India

**Keywords:** Anteversion, neck shaft angle, femoral torsion, coxa antetorsa, femur

## Abstract

Precise anatomical assessment of femoral neck anteversion (FNA) and the neck shaft angles (NSA) would be essential in diagnosing the pathological conditions involving hip joint and its ligaments. The present study was undertaken on 48 fetal femurs to calculate the NSA and FNA in fetuses digitally.

End on images of upper end of the femurs were taken for the estimation of FNA and a photograph in a perpendicular plane was taken to calculate the NSA. Microsoft Paint software was used to mark the points and Image J software was used to calculate the angles digitally.

The FNA ranged from 17.08º to 33.97 º on right and 17.32 º to 45.08 º on left. The NSA ranged from 139.33 º to 124.91 º on right and 143.98 º to 123.8 º on left. Unpaired t test showed the FNA and NSA of femur did not vary significantly during the third trimester.

## Introduction

Femoral neck anteversion (FNA) is represented by the angle between the longitudinal axis of the neck of - femur and the axis passing horizontally through femoral condyles^1^. depicts the degree of rotation of the femoral neck in reference to the coronal plane^2^.

The FNA is a result of fetal development, heredity, mechanical forces, and intrauterine position^3^. The value of FNA angle at birth is commonly about 40 degrees and decreases gradually to approximately 20 degrees by the age of ten, to finally achieve value around 8 to 15 degrees in adulthood^1, 2, 4-6^.

It is believed that physiological decline in FNA in children is influenced by dynamic forces produced during upright walking. Therefore, the magnitude of the angle is attributed to appropriate motor control, muscle balance and ligament-integrity^7^.

FNA of greater than 20 degrees is considered excessive femoral anteversion, whereas a torsion angle of less than 10 degrees is considered femoral retroversion^8,9^. Excessive femoral torsion is not uncommon and has been associated with certain neurologic and orthopedic conditions. For example, children with cerebral palsy have a high prevalence of excessive FNA.

In normal hips the neck-shaft angle ((NSA) should cause the longitudinal axes of the femoral necks to cross at the point of body weight. The enlargement and maturation of the hip joint increases at 20th week of gestation and the NSA ranges from 135- 140◦ at birth^10^.

These two angles (FNA and NSA) are associated with the pathology in newborns, children and adults. The most common femoral torsion abnormality is known as coxa antetorsa (excessive anteversion)^11^. Precise anatomical assessment of these angles would be essential in diagnosing the pathological conditions involving hip joint and its ligaments. Very limited literature is available on these angles in the Indian population.

Even though a lot of studies are available on these angles, very limited literature is available on the Indian population. Therefore the present study was aimed to calculate the NSA and FNA in fetuses digitally in the South Indian population. This describes the developmental changes in the hip joint in relation to these angles and also adds the information to the available literature.

## Materials and Methods

The present cross sectional study was carried out in the department of Anatomy, Kasturba Medical College, Manipal. Twenty-four (20 males, 4 females) formalin fixed fetuses of second and third trimesters with known gestational age (GA) were included in the study. The GA ranged from 13 to 36 weeks. The spontaneously aborted and stillborn fetuses were obtained from the department of Obstetrics and Gynecology after taking informed consent from the persons concerned. Ethical approval for the study was obtained from the Institutional Ethics Committee (IEC). The fetuses with any kind of gross external deformity were excluded from the study.

### Study design

The femurs were dissected bilaterally and the excess soft tissue was cleared. The femurs which were damaged during the dissection were excluded from the study. The femurs were tagged according to the GA and two photographs for each femur were taken using Nikon digital camera. Each femur was kept on a horizontal hard desk and end on image of the upper end was taken for the estimation of FNA and a photograph in a perpendicular plane was taken to calculate the NSA.

### Calculation of angles:

The images were transferred to the computer and were stored in TIFF format. Microsoft Paint software was used to mark the points and Image J software was used to calculate the angles digitally.

1. Calculation of FNA: The midpoint of the femoral head and the neck were marked and a line was drawn joining these two points. The angle made by this line with the horizontal plane was measured as FNA (Fig 1).

**Fig. 1:**
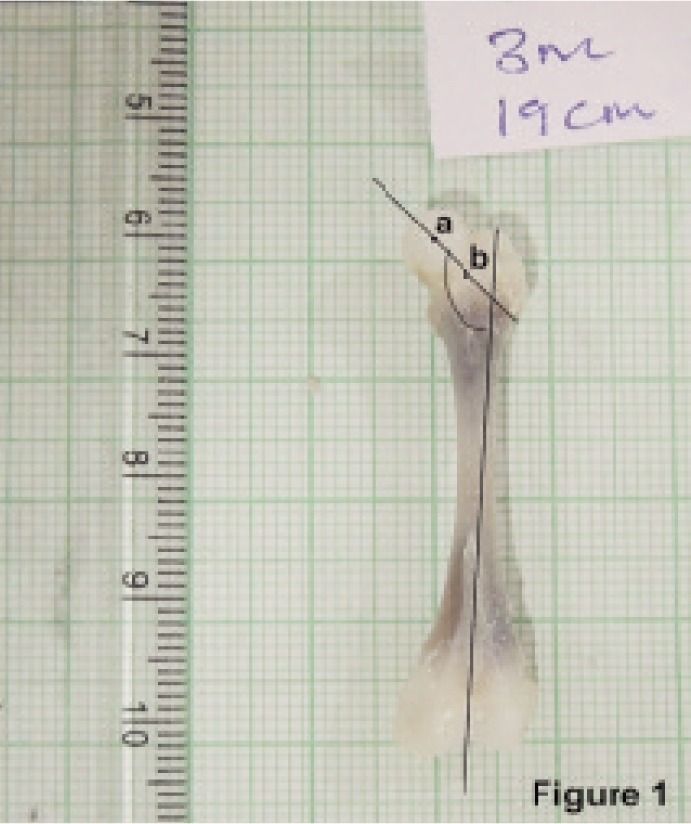
showing the perpendicular view of the fetal femur for measuring the NSA. a-Midpoint of the head, b- midpoint of the neck
2. Calculation of NSA: The midpoint of femoral head and the neck were marked and a line was drawn joining these two points. A vertical line was drawn passing through the tip of the greater trochanter to the inter-condylar notch. The angle formed by these lines was calculated as NSA (Fig 2).

**Fig. 2:**
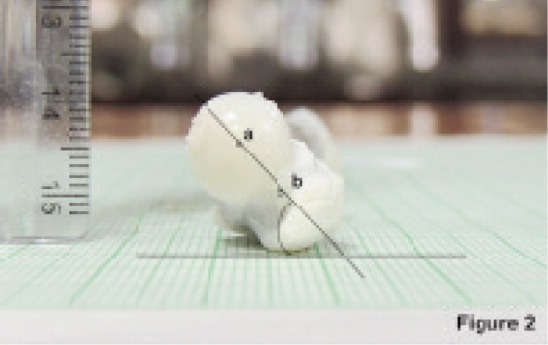
showing the end on view of the upper end of fetal femur for measuring the FNA. a-Midpoint of the head, b- midpoint of the neck

### Inter-observer repeatability:

The angles were measured by one observer and the data was stored in Microsoft Excel format. The same parameters were repeated by the second observer using the same software. Paired t-test was applied to compare the measurements from two different observers.

## Results

The present study was carried out using 48 fetal femurs (24 right, 24 left). The paired t-test for the inter-observer variability showed no significant difference between the values obtained by the two observers. (FNA: t=-1.06 and p=0.31; NSA: t=-0.09 and p=0.9).

There were 16 fetuses of second and 8 fetuses of third trimester. The mean and standard deviation (SD) of the angles measured are shown in table I. The FNA ranged from 17.08º to 33.97 º on right and 17.32 º to 45.08 º on left. The NSA ranged from 139.33 º to 124.91 º on right and 143.98 º to 123.8 º on left.

**Table I:** Mean and SD of FNA and NSA at second and third trimesters

Measured angle	Side	Number of specimens (N)	Second trimester	Third trimester
FNA	Right	16	24.82±6.27º	28.16±3.04º
	Left	16	26.64±8.14º	27.58±2.25º
NSA	Right	8	134.21±3.91º	132.21±5.56º
	Left	8	132.68±7.09º	129.41±3.87º

Unpaired t-test was applied to compare the mean angles between the second and third trimesters which did not show any statistical significance (p=0.2 for FNA and p=0.56 for NSA). This shows that the FNA and NSA of femur did not vary significantly during the third trimester.

The FNA and NSA were correlated with the GA using Pearson’s correlation. FNA appeared to increase with the GA (r=0.5) but the increase was not statistically significant (p=0.1). A decrease in the NSA was also noted with the GA (r=-0.3) which was also statistically not significant (p=0.28).

## Discussion

It was known that the FNA and NSA change during childhood, until growth is completed^12^. Between three and twelve months of age, the FNA is 39º, reaching adult life with a value close to 16º. Determining the FNA value is crucial for the diagnostic and therapeutic planning of patients with various pathologies, such as hip development dysplasias, cerebral palsy, varum thigh, flat thigh, epiphysiolysis, congenital club foot and other developmental and metabolic abnormalities^13^.

Jouvea *et al* studied the FNA and NSA of fetal femurs using 45 fetuses (16 females and 28 males). Photoshop 7.0 software was used for the angle estimation. It was found that the FNA and NSA were both correlated with age (FNA increases with age; NSA decreases with age). There was no correlation between FNA or NSA and sex. These two angles were associated with pathology in newborns, children and adults^14^.

FNA was studied by Upadhyay *et al* in two groups of adults, one group previously suffered from traumatic posterior dislocation of the hip, and the other group was normal adult volunteers. FNA on both the injured and uninjured side was significantly reduced in the patients when compared to the volunteers^15^.

Adrian, in his study on 24 cadaver fetuses (14 female and ten male specimens) by radiograph method observed that the mean NSA was 119.8±26.2º. The FNA ranged from 15 to 33 degrees^16^.

A fetal study by Andrzej et al on 106 human fetuses (16 to 38 weeks) using a ‘FEM-GEO_03’ computer Program, found that the mean and SD of NSA was 140.48±6.95°. No significant differences were found in NSA between male and female fetuses or between left and right bones. Dispersion analysis showed a decrease in the NSA angle during fetal development, which suggests that the adaptation to a vertical position and bipedal gait starts during pregnancy and is manifest as an inborn feature^17^.

In the present study The FNA ranged from 17.08º to 33.97 º on right and 17.32 º to 45.08 º on left. The NSA ranged from 139.33 º to 124.91 º on right and 143.98 º to 123.8 º on left. The FNA and NSA of femur did not vary significantly during the third trimester. The study also revealed that the FNA increased with the GA whereas the NSA decreased. But this change was statistically not significant.

Decrease in the FNA angles was observed from birth till the end of growth process^1, 4, 18^. Few authors found that he FNA increased during the second half of gestation. The author explained that those changes may have been caused by mechanical stresses. This could explain the positive correlation between age and FNA^19, 20^. In opposition to FNA angle, NSA is more stable, not changing significantly, either during the second or the third trimester of pregnancy^14^.

A study done by Kornelia *et al* in twenty-eight adults (4 men, 24 women) observed the average angles of FNA measured by ultrasound and by MRI were 20.7±11.0º and 19±11.3º respectively^21^.

A database of FNA and NSA was measured using fluoroscopic method in 147 patients with cerebral palsy. The angles of FNA were similar at early ages between healthy children and children with cerebral palsy. As the age of the children increased, those with cerebral palsy showed little change in FNA, whereas the healthy children had progressively decreasing angles of FNA as they approached adulthood. The NSA increased significantly in children with cerebral palsy when compared to the angles of healthy children^20^.

The gender differences were not considered in the present study due to the small number of female fetuses available. The smaller number of fetuses in the third trimester could be one of the reasons for not getting a significant difference in the angles between second and third trimesters.

## Conclusion

The present study evaluates the FNA and NSA of femur in fetuses and correlates their development with the GA during second and third trimesters. The FNA and NSA did not vary significantly during the third trimester. The further changes in their values in later life would be due to the mechanical stress factors involved that could modify a primary anatomical shape.
